# Collagen-Glycosaminoglycan Matrix Implantation Promotes Angiogenesis following Surgical Brain Trauma

**DOI:** 10.1155/2014/672409

**Published:** 2014-09-17

**Authors:** Kuo-Feng Huang, Wei-Cherng Hsu, Jong-Kai Hsiao, Gunng-Shinng Chen, Jia-Yi Wang

**Affiliations:** ^1^Division of Neurosurgery, Department of Surgery, Taipei Tzu Chi Hospital, Buddhist Tzu Chi Medical Foundation, New Taipei City 23143, Taiwan; ^2^School of Medicine, Tzu Chi University, Hualien 97004, Taiwan; ^3^Department of Ophthalmology, Taipei Tzu Chi Hospital, Buddhist Tzu Chi Medical Foundation, New Taipei City 23143, Taiwan; ^4^Department of Radiology, Taipei Tzu Chi Hospital, Buddhist Tzu Chi Medical Foundation, New Taipei City 23143, Taiwan; ^5^Graduate Institute of Medical Sciences, College of Medicine, Taipei Medical University, 250 Wu-Xing Street, Taipei 110, Taiwan

## Abstract

Surgical brain injury (SBI) is unavoidable during many neurosurgical procedures intrinsically linked to postoperative neurological deficits. We have previously demonstrated that implantation of collagen glycosaminoglycan (CG) following surgical brain injury could significantly promote functional recovery and neurogenesis. In this study we further hypothesized that this scaffold may provide a microenvironment by promoting angiogenesis to favor neurogenesis and subsequent functional recovery. Using the rodent model of surgical brain injury as we previously established, we divided Sprague-Dawley male rats (weighting 300–350 g) into three groups: (1) sham (2) surgical injury with a lesion (L), and (3) L with CG matrix implantation (L + CG). Our results demonstrated that L + CG group showed a statistically significant increase in the density of vascular endothelial cells and blood vessels over time. In addition, tissue concentrations of angiogenic growth factors (such as VEGF, FGF2, and PDGF) significantly increased in L + CG group. These results suggest that implantation of a CG scaffold can promote vascularization accompanied by neurogenesis. This opens prospects for use of CG scaffolds in conditions such as brain injury including trauma and ischemia.

## 1. Introduction

A criterion for tissue engineering scaffold to replace or restore the function of damaged tissues after surgical trauma is to mimic the complexity and microarchitecture of biological tissues, particularly the formation of functional vascular structures [1]. Our previous study suggests that applying collagen-glycosaminoglycan (CG) scaffolds not only provides a physical support structure but also promotes functional recovery and neurogenesis after surgical brain injury [2].

Promoting both angiogenesis, the formation of new capillaries from preexisting blood vessels, and neurogenesis, the formation of new nerve tissues, is therefore crucial when developing a specific cerebral microenvironment. Subsequent neuronal regeneration depends on a combination of angiogenesis and neurogenesis, which means that any microenvironment for developing the vasculature must also be accompanied by the proliferation and differentiation of neural precursor cells [3].

It is known that vascularization is of critical importance to tissue engineering, and, without it, the development of clinically applicable replacement tissue is extremely limited. Angiogenesis is the primary mechanism for vascularization of engineered biomaterials [4]. Neurogenesis and angiogenesis may play important roles in mediating functional recovery following experimental traumatic brain injury [5].

The aim of this work was to evaluate the potential of CG scaffolds as a biodegradable platform for coupling neurogenesis and angiogenesis and to further understand the role played by collagen-GAG and how its cellular and molecular microenvironments influence angiogenesis. This report describes the potential of collagen-GAG for use in promoting angiogenesis accompanying neurogenesis following surgical brain trauma.

## 2. Materials and Methods

### 2.1. CG Preparation and Assessment

**Figure 1:**
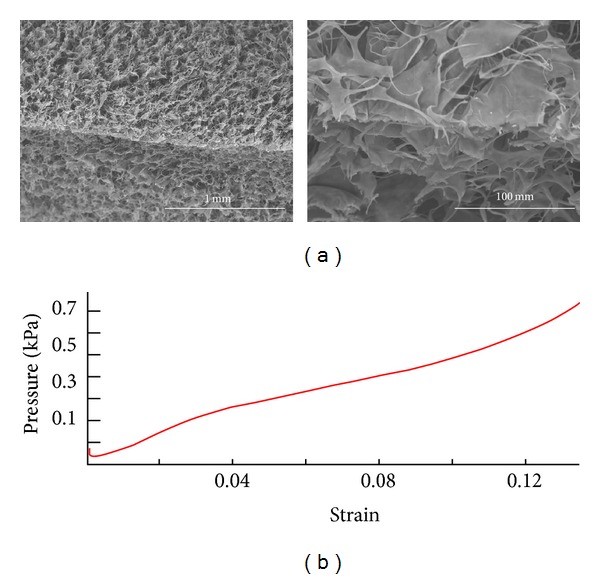
Characteristics of the collagen-glycosaminoglycan matrix. (a) Illustration of scanning electronic microscopy showing the porous structure, with pores at 20~200 *μ*m in diameter. Scale bar in left represents 1 mm, in right represents 100 *μ*m. (b) Pressure-strain interaction of the CG.

We produced a biodegradable collagen matrix of 1% collagen/0.02% GAG copolymer as previously described with modifications [6–8]. In brief, type I CG was prepared as a weak acidic aqueous solution and was stirred at high speed to form a slurry-like composition. After lyophilization, it was cross-linked via thermal dehydration in a vacuum, followed by exposure to UV light [9]. The C-GAG copolymer matrix scaffold was cut into blocks of 6 × 4 × 3 mm (72 mm^3^) before implantation, and we designated an optimal degradation time of around 28 days, in step with the time course of endogenous neural stem cell (NSC) proliferation and differentiation [10]. We also coated selected sections of dehydrated CG with 5 nm of gold in an Emitech K550 sputter-coater and examined the microstructures under a scanning electron microscope (S-360, Cambridge Instruments, Cambridge, UK) (Figures 1(a) and 1(b)). The biomaterial characteristics of the CG were then assessed by the strain-pressure interaction (Figure 1(b)). We measured the change in height of a CG following gradual application of an external force. A change in matrix height/original matrix height was defined as the strain [11]. When the CG was compressed by external pressure, its height was gradually reduced. During application of the increased external force on the matrix, the strain gradually increased (Figure 1(b)) [12].

### 2.2. Animal Model of Surgically Induced Traumatic Brain Injury and Designation of the Experimental Groups

**Figure 2:**
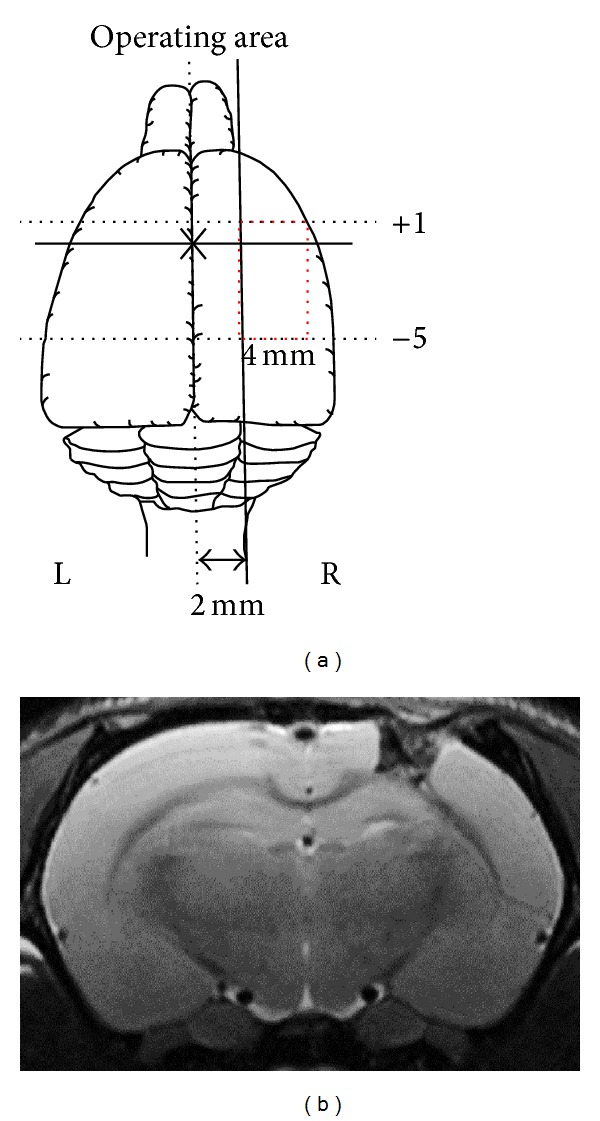
Schematic drawing of the experimental surgical brain trauma model and MRI demonstration. (a) Illustration of a structural drawing demonstrating the rat brain cut along a horizontal plane and showing surgical trauma to the frontal lobe in relation to the bregma. (b) Demonstration by MRI of surgical brain trauma with implantation of a collagen-glycosaminoglycan matrix. The exact location of the graft was visualized in vivo by MR T2-weighted images as a hypointense area located in the left frontal cortex, which matches the intended surgical implantation area.

All of the procedures involving animals followed the* Guiding Principles in the Care and Use of Animals* of the American Physiology Society and were approved by the Animal Care and Use Committee of Taipei Tzu Chi Hospital, Buddhist Tzu Chi Medical Foundation. Adult male Sprague-Dawley rats weighing 300~350 g were anesthetized by an intraperitoneal injection of pentobarbital (65 mg/kg) and placed in a prone position in a stereotaxic apparatus. Surgery was performed under sterile conditions. Animals were placed into one of the following groups. (1) Animals in the sham (SHAM) group received only a craniotomy and replacement of the bone flap. (2) In the lesion (L) group, after a craniotomy and exposure of a square window displaying the underlying right frontal lobe of the brain covered by the dura, the dura was carefully incised with a number 20 needle to minimize bleeding, and a frontal parietal lesion (6 × 4 mm edge) was localized from 1.0 mm anterior to 4.0 mm posterior of the bregma and 1.0 to 5.0 mm lateral to the midline. The depth of the lesion was 3.0 mm from the brain surface. (3) In the lesion + CGM (L + CGM) group, after the procedure for the L groups was carried out, a block of CGM scaffold was placed into the lesion cavity formed following tissue removal from the frontal parietal area (Figure 2(a)). The number in each group was 10 (*n* = 10). All procedures used sterile gauze pads and saline irrigation to control bleeding, and the skin was sutured using 3-0 silk (Ethicon, Taiwan, Taipei). We monitored vital signs throughout the procedures.

### 2.3. In Vivo Magnetic Resonance Imaging (MRI) Protocol

After implantation of the CG scaffold, a clinical 1.5-T MRI system was used to observe the morphology of the CG scaffold in vivo. Under gas anesthesia with 2% isoflurane, a mouse was placed in a homemade resonance coil with an inner diameter of 3.7 cm. Fast spin echo pulse sequences provided by the vendor were used (TR/TE of 4000/101.4 ms). The slice thickness was 0.8 mm with a 0.2 mm gap, and the field of view (FOV) was 5 × 2.5 cm. The total scan time was 3 min and 20 s at an NEX of 8. Images were then analyzed at a workstation provided by GE Healthcare [12]. We verified the exact location of the graft in living animals by MRI. On a T2-weighted image, the shape of the graft in the left frontal cortex was observed to be preserved as a hypodense rectangular area, which was the best direct evidence of successful implantation of the scaffold (Figure 2(b)).

### 2.4. Tissue Preparation for Histology

Rats were anesthetized and perfused transcardially with phosphate buffered saline (PBS) followed by 4% paraformaldehyde on D7, D14, D21, and D28. Brains were removed then fixed in 4% paraformaldehyde overnight and embedded in paraffin blocks. Serial sections (6 *μ*m) through the cerebral cortex were processed for double immunofluorescence staining.

### 2.5. Double Immunofluorescence Staining

For antigen retrieval, brain sections were treated with 50% formamide, 280 mmol/L NaCl, and 30 mmol/L sodium citrate at 65°C for 2 hours, incubated in 2 mol/L HCl at 37°C for 30 minutes, and rinsed in 0.1 mol/L boric acid (pH 8.5) at room temperature for 10 minutes. Sections were blocked in PBS containing 1% goat serum, 0.3% triton X-100 for 1 hour and followed by incubation with primary antibodies, either (1) a mouse monoclonal anti-NeuN antibody (Millipore; 1 : 1000, Temecula, CA) and a rabbit polyclonal anti-Ki-67 antibody (AnaSpec; 1 : 500, San Jose, CA), (2) a mouse monoclonal anti-*α* smooth muscle actin (SMA) antibody (Sigma; 1 : 500, St. Louis, MO) and a rabbit polyclonal anti-RECAM-1(CD31) antibody (Millipore; 1 : 500), (3) a mouse monoclonal anti-*α*-SMA antibody (Sigma; 1 : 500) and a rabbit polyclonal anti-Ki67 antibody (AnaSpec; 1 : 500), or (4) a rabbit polyclonal anti-RECAM-1(CD31) antibody (Millipore; 1 : 500) and a mouse monoclonal anti-endothelial cell (RECA-1) antibody (Abcam; 1 : 50, Cambridge, UK) at 4°C overnight and with secondary antibodies (Alexa Fluor, 488 goat anti-mouse immunoglobulin G (IgG; 1 : 200, Invitrogen, Carlsbad, CA) and DyLight 549 anti-rabbit IgG (1 : 200 dilution, Jackson ImmunoResearch, West Grove, PA)) at room temperature for 2 h. Sections were mounted with mounting medium H-1000 (Vector Laboratories, Burlingame, CA). Nonspecific staining was visualized by omitting the primary antibody and was negative. Fluorescent microscopic images were obtained with a Nikon ellipse 80i microscope (Nikon Optical, Tokyo, Japan) and a Nikon Digital Sight DS-5 M camera using NIS-Elements F 2.30 software (Nikon). Digital image processing was performed with Image-pro Plus, version 5.1.

### 2.6. Cell Counting

Following double immunofluorescence staining, numbers of positively stained cells in the intramatrix zone (IMZ) and lesion boundary zone (LBZ) on day 7 (D7), D14, D21, and D28 were counted manually in three to five different fields per section of each rat brain (using an eyepiece grid covering an area of 0.0625 mm^2^) by an individual who was blinded to the experimental design. Vessels and blood cells were excluded. Sections were observed, and images were acquired using a Nikon epifluorescent microscope. Total cells in the IMZ and LBZ were visualized by DAPI staining at 20x magnification using Openlab software (Improvision, Cambridge, MA). Ki67^+^ cells were visualized as fluorescent red (DL 549), and NeuN^+^ cells were visualized as fluorescent green (Alexa 488) in the IMZ and LBZ at 20x, while double-positive cells were visualized as yellow. For double immunofluorescence staining, double-positive cells were also manually counted in the same manner. Finally, we present the results as the number of immunopositive cells per field.

### 2.7. Enzyme-Linked Immunosorbent Assay (ELISA) for Measurement of the Tissue Concentrations of Platelet-Derived Growth Factor (PDGF), Fibroblast Growth Factor-2(FGF2), and Vascular Endothelial Growth Factor (VEGF)

Brains from the sham, L, and L + CG groups of animals were removed after cervical dislocation on D7, D14, D21, and D28 after surgery. A 3 mm coronal section was taken from the injured area over the parietal cortex, snap-frozen in liquid nitrogen, and stored at −70°C until used. All brain samples were homogenized in buffer consisting of 0.05 M Tris HCl, 0.15 M NaCl, 0.1% Nonidet 40, 0.5 M phenylmethylsulfonyl fluoride, 50 mg/mL aprotinin, 10 mg/mL leupeptin, 50 mg/mL pepstatin, 4 mM sodium orthovanadate, 10 mM sodium fluoride, and 10 mM sodium pyrophosphate. Homogenates were centrifuged at 4°C and 12,000 g for 15 min. Then the supernatants were removed and assayed in duplicate using VEGF and PDGF assay kits (R&D Systems, Minneapolis, MN) and a FGF2 assay kit (MyBioSource, CA) according to the manufacturer's guidelines. Concentrations of tissue proteins (VEGF, PDGF, and FGF2) were expressed as picograms of antigen per milligram of protein. In our experiments, the concentration of VEGF from tissue samples was measured on D7, D14, D21, and D28 with an ELISA kit.

### 2.8. Statistical Analysis

Comparisons between multiple groups were conducted using a one-way analysis of variance (ANOVA) with the post hoc Bonferonni *t*-test for multiple comparison. All statistical analyses were performed using Sigma Stat version 2.0 from Jandel Scientific (San Diego, CA). Data are expressed as the mean ± standard deviation (SD). Differences were considered significant as *P < 0.05, **P < 0.01, and ***P < 0.001 for L or L + CG groups versus the sham group; ^+^ *P* < 0.05, ^++^ *P* < 0.01, and ^+++^ *P* < 0.001 for L + CG versus L.

## 3. Results

### 3.1. Increased Proliferating Neurons Cells within the Lesion Boundary Zone (LBZ) in the L + CG Group

**Figure 3:**
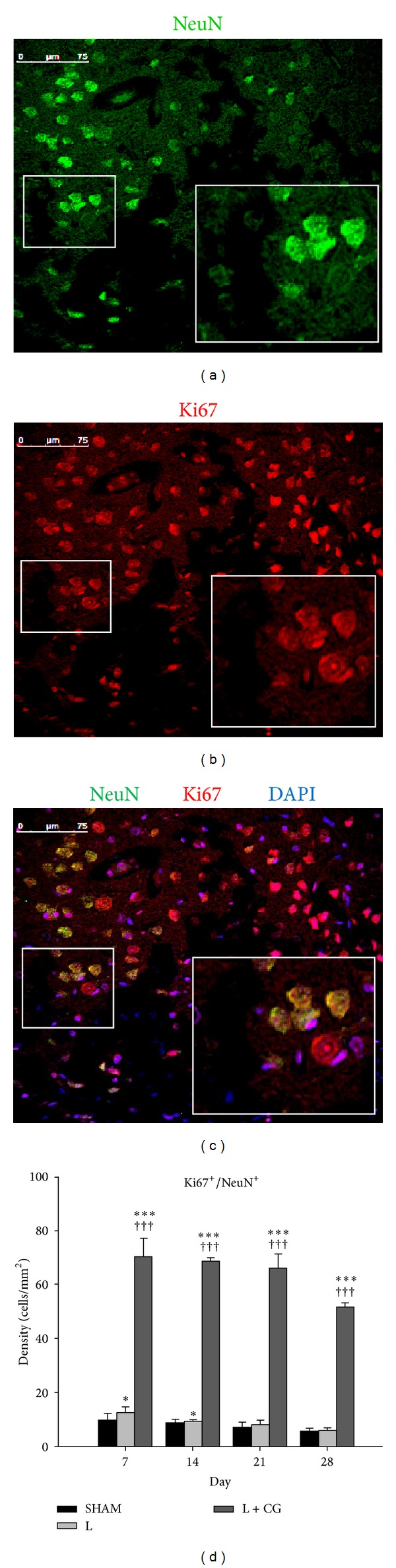
Proliferating neurons defined with NeuN^+^/Ki67^+^ immunoreactivity in the lesion boundary zone (LBZ) of a surgical traumatic brain lesion with implantation of a collagen-glycosaminoglycan matrix on day 14. Demonstrative photomicrographs of double immunofluorescence staining of illustrative brain sections from L+CG rats on day 14 following surgical trauma. (a) Immunoreactivity of NeuN (green), a neuronal marker, and (b) Ki67 (red), which stains proliferating cells in the intra-matrix zone (IMZ) of the L+CG group. DAPI (diamidino-2-phenylindole) (blue), a classic nuclear counterstain for immunofluorescence, and merged image (c). Scale bars in (a–c) represent 75 *μ*m. (d) Numbers of proliferative neurons (Ki67^+^/NeuN^+^) from brains of the L+CG group on D7, D14, D21 and D28 after surgery in the intra-matrix zone (IMZ). Data are the mean ± SD. *P < 0.05, **P < 0.01, and ***P < 0.001, L and L+CG groups versus the Sham group; ^+^ *P* < 0.05, ^++^ *P* < 0.01 and ^+++^ *P* < 0.001, L versus L+CG.

Representative photomicrographs show double immunofluorescence staining with antibodies against Ki-67 (red, a cellular marker of proliferation), and NeuN (green, a neuronal marker) of brain sections from L + CG rats on D14 following injury. Further, we counted the density (cells/mm^2^) of NeuN^+^/Ki67^+^ cells in the LBZ of the sham, L, and L + CG groups of rats at various time points. The L + CG group also showed a significant increase in NeuN^+^/Ki67^+^ cells on D7 after the surgical brain lesion, with levels sustained and peaking with a slight increase on D28. CG implantation promoted proliferation of neurons as defined by NeuN^+^/Ki67^+^ immunoreactivity in the LBZ of surgical brain lesions after implantation. See Figure 3.

### 3.2. Increased Proliferating Smooth Muscle Cells in Vessel Walls in the LBZ of the L + CG Group

**Figure 4:**
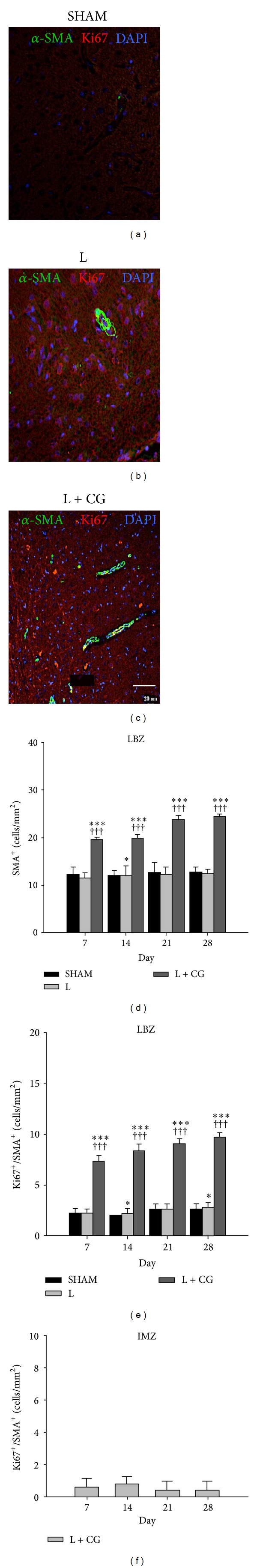
Proliferating smooth muscle cells in vessel walls defined with *α*-smooth muscle actin (*α*-SMA)^+^/Ki67^+^ immunoreactivity in the lesion boundary zone (LBZ) of surgical traumatic brain lesions with implantation of a collagen-glycosaminoglycan matrix on day 21 (D21). Representative photomicrographs (merged images) of immunofluorescence staining of representative brain sections from Sham (a), lesion (L) (b) and L+CG (c) rats on day 21 following surgical trauma. Immunoreactivity of *α*-SMA (green), which stains smooth muscle cells in vessel walls, and Ki67 (red), which stains proliferating cells. DAPI (diamidino-2-phenylindole) (blue), a classic nuclear counterstain for immunofluorescence. Scale bars in (a–c) represent 75 *μ*m. Numbers of *α*-SMA^+^ (d), proliferative smooth muscle cells in vessels walls (Ki67^+^/*α*-SMA^+^) (e) from brains of the L+CG group on D7, D14, D21 and D28 after surgery in the lesion boundary zone (LBZ) and proliferative smooth muscle cells in vessels walls (Ki67^+^/*α*-SMA^+^) (f) intra-matrix zone (IMZ). Data are the mean ± SD. *P < 0.05, **P < 0.01, and ***P < 0.001, L and L+CG groups versus the Sham group; ^+^ *P* < 0.05, ^++^ *P* < 0.01 and ^+++^ *P* < 0.001, L versus L+CG.

*α*-SMA is commonly used as a marker of myofibroblast formation [13]. To identify if the existence of proliferative smooth muscle cells was correlated with angiogenesis, we examined the density of *α*-SMA^+^/Ki67^+^ cells within the LBZ. Double immunofluorescent staining of *α*-SMA^+^/Ki67^+^ cells identified double-positive cells (proliferative smooth muscle cells) in the IMZ and LBZ (Figure 4). We also measured the density (cells/mm^2^) of *α*-SMA^+^/Ki67^+^ cells in the LBZ of the sham, L, and L + CG groups of rats at various time points. The L + CG group also showed a significant increase in *α*-SMA^+^/Ki67^+^ cells on D7 after the surgical brain lesion, with levels sustained and peaking with a slight increase on D28. We also found some *α*-SMA^+^/Ki67^+^ cells existed in the IMZ of the L + CG group.

### 3.3. Increased Endothelial Cells and Smooth Muscle Cells in the IMZ and LBZ with Implantation of CG Scaffolds

**Figure 5:**
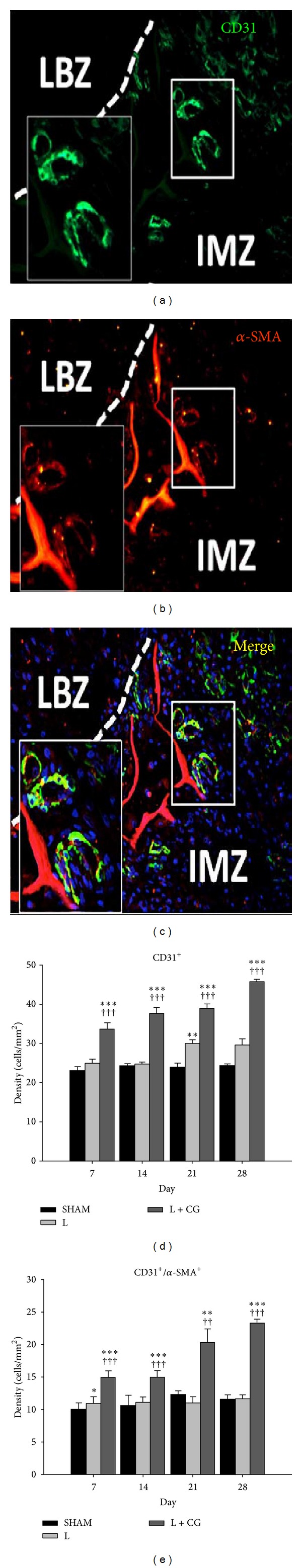
Both endothelial cells and smooth muscle cells in vessel walls defined by CD31/*α*-smooth muscle actin (*α*-SMA)^+^ immunoreactivity in the intra-matrix zone (IMZ) of surgical traumatic brain lesions with implantation of a collagen-glycosaminoglycan matrix on day 21 (D21). (a) Immunoreactivity of CD31 (green), an endothelial cell marker, and (b) *α*-SMA (red), which stains smooth muscle cells in vessel walls in the intra-matrix zone (IMZ) of the L+CG group. DAPI (diamidino-2-phenylindole) (blue), a classic nuclear counterstain for immunofluorescence, and merged image (c). (d) Numbers of endothelial cells (CD31^+^), (e) CD31^+^/*α*-SMA^+^ cells from brains of Sham, L, and L+CG groups on D7, D14, D21, and D28 after surgery in the LBZ. Data are the mean ± SD. *P < 0.05, **P < 0.01, and ***P < 0.001, L and L+CG groups versus the Sham group; ^+^ *P* < 0.05, ^++^ *P* < 0.01 and ^+++^ *P* < 0.001, L versus L+CG.

To evaluate angiogenesis, we used CD31 which is mainly used immunohistochemically to define the presence of endothelial cells [14]. The earliest marker common to both endothelial and hematopoietic precursors so far identified is CD31 [15]. We verified that lumens with simultaneous CD31 and *α*-SMA staining were arterioles. Isolated cells that stained positive for *α*-SMA with or without elongating transformation were regarded as myofibroblasts [11]. We also examined whether CD31^+^/*α*-SMA^+^ cells existed in the IMZ and LBZ of surgical traumatic brain lesions after implantation of the CG scaffold. Double immunofluorescent staining of CD31^+^/*α*-SMA^+^ cells allowed recognition of double-positive cells (both endothelial cells and smooth muscle cells in vessel walls) in the IMZ of surgical traumatic brain lesions with implantation of the CG (Figure 5). We also measured the density (cell/mm^2^) of CD^+^ cells and CD31^+^/*α*-SMA^+^ cells in the LBZ of the sham, L, and L + CG groups of animals at various time points. The L + CG group also showed significant increases in both CD31^+^ cells and CD31^+^/*α*-SMA^+^ cells in the LBZ on D7 with a sustained increase up to D28 (Figures 5(d) and 5(e)).

### 3.4. Increased Proliferative Endothelial Cells in the LBZ after CG Implantation

**Figure 6:**
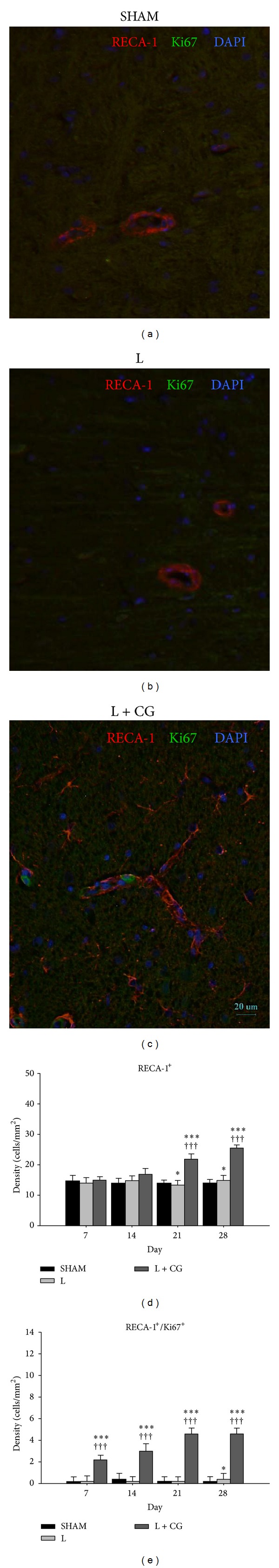
Proliferative endothelial cells defined by anti-endothelial cell antibody (RECA)1^+^/Ki67^+^ immunoreactivity in the lesion boundary zone (LBZ) of surgical brain trauma with collagen-glycosaminoglycan matrix implantation on day 14 (D14). Representative photomicrographs (merged images) of immunofluorescence staining of representative brain sections from Sham (a), L (b) and L+CG (c) rats on D14 following surgical trauma. Immunoreactivity of RECA1 (red) which stains endothelial cells, and Ki67 (green), which stains proliferating cells. DAPI (diamidino-2-phenylindole) (blue), a classic nuclear counterstain for immunofluorescence. Scale bars in (a–c) represent 75 *μ*m. (d-e) Numbers of RECA1^+^ cells (which represents endothelial cells), Ki67^+^ cells (proliferative cells), and RECA1^+^/Ki67^+^ (proliferative endothelial cells) from brains of the Sham, L, and L+CG groups on D7, D14, D21, and D28 after surgery in the LBZ. Data are the mean ± SD. *P < 0.05, **P < 0.01 and ***P < 0.001, Lesion (L) and L+CG groups versus Sham group; ^+^ *P* < 0.05, ^++^ *P* < 0.01 and ^+++^ *P* < 0.001, L versus L+CG.

To further identify proliferative endothelial cells in the LBZ, we instituted double immunofluorescent staining of RECA-1^+^/Ki67^+^ cells to confirm whether angiogenesis existed. RECA-1 is a well-known endothelial cell antibody in rat studies and is especially used to further identify the character and function of endothelial cell-specific antigens [16]. Double immunofluorescent staining of RECA-1^+^/Ki67^+^ cells identified double-positive cells (proliferative endothelial cells) in the IMZ and LBZ (Figure 6). We further measured the density (cells/mm^2^) of RECA-1^+^/Ki67^+^ cells in the LBZ of the sham, L, and L + CGM groups of rats at different time points. The L + CG group also showed a significant increase in RECA-1^+^/Ki67^+^ cells on D7 after the surgical brain lesion, although significant increases in RECA-1^+^ cells, both RECA-1^+^ and RECA-1^+^/Ki67^+^ cells, were shown with sustained increases in levels.

### 3.5. Increased Tissue Concentrations of FGF2, PDGF, and VEGF

**Figure 7:**
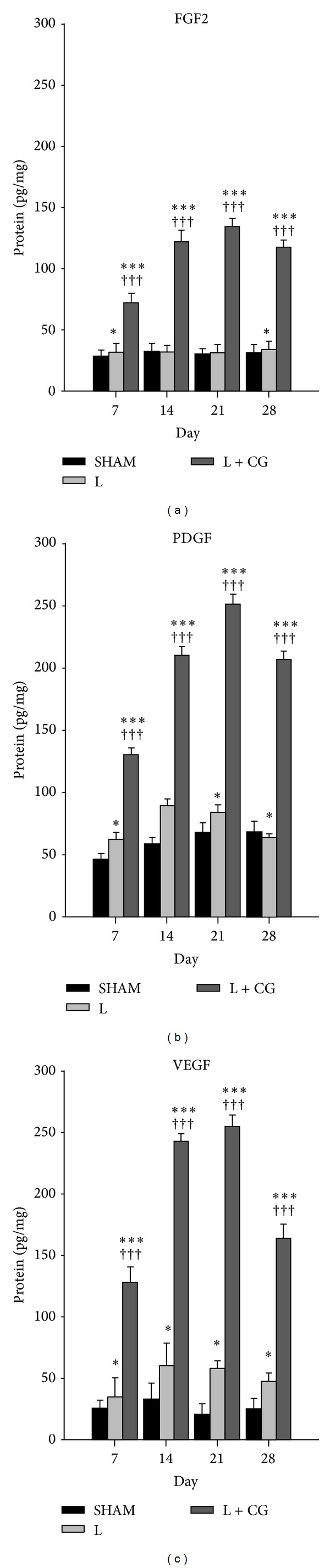
Sustained increase of tissue concentrations of angiogenesis growth factors fibroblast growth factor- (FGF-2), platelet-derived growth factor (PDGF), and vascular endothelial growth factor (VEGF) in the lesion boundary zone (LBZ) of brains from of L+CG group compared to those from the Sham or L groups. (a) FGF2, (b) PDGF, and (c) VEGF protein concentrations as measured by an ELISA in brains of the Sham, L, and L+CG groups of rats. Data are the mean ± SD. *P < 0.05, **P < 0.01 and ***P < 0.001, L and L+CG groups versus Sham group; ^+^ *P* < 0.05, ^++^ *P* < 0.01 and ^+++^ *P* < 0.001, L versus L+CG.

We further measured tissue concentrations of FGF2, PDGF, and VEGF in the LBZ of the sham, L, and L + CG group rats on D7, D14, D21, and D28 after the surgical brain lesion. The L + CG group showed significant increases (all *P* < 0.001) in tissue concentrations of FGF2, PDGF, and VEGF as early as D7 (*F*(2, 12) = 72.84, 107.63, 386.78, resp.); and all exhibited peak levels on D21 (*F*(2, 12) = 476.94, 818.76, 1127.37, resp.) and sustained to D28 (*F*(2, 12) = 185.63, 672.47, 333.75, resp.). In addition to significant increases compared to sham group (all *P* < 0.001), post hoc analysis (Bonferroni multiple comparison) also indicated significant increases (all *P* < 0.001) in tissue concentrations of FGF2, PDGF, and VEGF in L + CG group compared to L group at D7, D14, D21, and D28 (Figure 7).

## 4. Discussion

In the present study we have demonstrated promotion of angiogenesis accompanied by neurogenesis after CG implantation in a rat surgical brain lesion model. We have also demonstrated histological findings of proliferative neurons in the LBZ of the L + CG group and proliferating smooth muscle cells (SMA^+^/Ki67^+^) in vessel walls in the LBZ of the L + CG group. We found an increase in endothelial cells (CD31^+^) after implantation of the CG scaffold following surgical brain trauma, and proliferative endothelial cells (CD31^+^/Ki67^+^) also significantly increased. Tissue concentrations of VEGF, FGF2, and PDGF also increased after CG scaffold implantation.

One essential reason for the lack of clinical success in forming viable vascular systems seems to be the complexity of the process of angiogenesis. Formation of new capillary vessels is initiated by certain growth factors. Degradation of the extracellular matrix (ECM) and surrounding parent capillary basement membrane can subsequently release proteinase enzymes, which leads to liberation of ECM-bound growth factors such as VEGF [17]. Our CG scaffold is an analog of the ECM and was previously found to have many applications in the biomedical domain due to its low antigenicity, suitable biodegradability, and good mechanical, hemostatic, and cell-binding features [18, 19]. It is also known that GAG promotes the binding and modulation of growth factors and cytokines and the inhibition of proteases and is involved in the adhesion, migration, proliferation, and differentiation of cells [20–22]. Furthermore, GAG is practically nonimmunogenic. In addition to collagen purity and matrix morphology, the chemical methods used for collagen crosslinking and attachment of GAG are essential characteristics; they also influence cellular reactions [23, 24]. In tissue engineering, vascularization of implanted scaffolds is often inadequate, and this seriously obstructs the survival of cells, which perish due to a lack of oxygen and nutrients, and insufficient removal of waste products. Consequently several approaches have been employed to increase the vascularization of engineered tissue constructs. For example, pore sizes of scaffolds were varied to find the optimal diameter for cellular adhesion and migration [25]. Furthermore, some reports included endothelial cells and fibroblasts in CG scaffolds so as to initiate angiogenesis in vitro [26]. Besides, some reports also proved that the addition of GAG and growth factors could increase angiogenesis in vivo [27]. The main concerns in our study were the degradation timing and pore size. We designed a degradation time of about 28 days and found that some growth factors related to vascularization simultaneously increased. Accompanied by increases in permeability and degradation of the surrounding matrix, activated and proliferating endothelial cells were found to migrate and differentiate. In this study, our CG scaffolds also seemed to play a critical role in promoting proliferation of periendothelial cells especially in the area surrounding the lesion.

Our previous report also showed that neurological functional recovery significantly improved following implantation of a CG after surgical brain trauma [2]. It was also reported that neurogenesis and angiogenesis are coupled processes. Neurogenesis is known to be controlled by some intrinsic genetic mechanisms, and the crucial process of migrating NPCs is strongly related to the proliferation of blood vessels, signifying that blood vessels must play an essential role as a scaffold for NPC migration toward the damaged brain region [28, 29].

It is known that many trophic factors have played roles of neuroprotection following neuronal injury. BDNF was known to play important neuroprotective roles through BDNF-TrkB signaling [30] and have beneficial effects of cell survival and synaptic plasticity [31]. Our previous study has shown that CG matrix implantation increased tissue concentration of BDNF following surgical brain trauma [2]. Other previous studies also show that transplantation of BDNF-hypersecreting human mesenchymal cells can improve functional outcome following acute spinal cord injuries [32].

Besides BDNF, VEGF which was originally known to be a specific angiogenic factor is now known to have significantly beneficial effect on neurogenesis after TBI [33]. Another report also shows that VEGF itself have direct neuroprotective effects on neurons [34]. However, the potential neuroprotective effect of VEGF on cortical neurons after mechanical trauma injury has not been clearly established. In addition, PDGF is known to be both angiogenic and neuronal survival factor which is also reported to be an important component of neurovascular crosstalk [35]. FGF2 is also known to be closely involved in neuronal protection and repair after ischemic, metabolic, or traumatic brain injury [36]. In the present study we observed significant increase of FGF2, PDGF, and VEGF in L + CG group. However, which trophic factor is mainly responsible and the detailed molecular mechanisms still need further investigation.

Angiogenesis is a multifactorial process regulated by many factors, and it is also known that the recruitment of periendothelial cells is mediated by many local growth factors. An important stimulating factor in angiogenesis is VEGF. The presence of VEGF allows endothelial cells to proliferate and migrate in the direction of the scaffold, where they begin proliferating and differentiating. FGF2 is also known for its angiogenic potential which stimulates endothelial cells to produce VEGF and increases VEGF receptor expression. FGF2 also stimulates endothelial cell migration, pericyte attraction, and matrix deposition [37]. Additionally, it was reported that PDGF is regarded as a chemoattractant for smooth muscle cells (which comprise the vessel walls of arterioles), while VEGF, possibly via release of PDGF or binding to VEGF receptors, also contributes to this effect [38]. Angiogenesis processes are regulated by various growth factors, and VEGF is the most potent in promoting the formation of new blood vessels [39, 40]. The need for multiple growth factors was investigated by Laschke et al. [41], who found that angiogenesis and blood vessel maturation can be suppressed by complete inhibition of either VEGF, FGF2, or PDGF. It is also known that VEGF contributes to the complexity of the vascular network by stimulating vascular splitting and sprouting [42]. We demonstrated that angiogenesis increases with increasing tissue concentrations of these growth factors. Our results emphasized that increased tissue concentrations of growth factors (VEGF, FGF2, and PDGF) may promote angiogenesis and result in a higher density of blood vessels [43]. It is recognized that growth factors contribute to tissue regeneration at various stages of cell proliferation and differentiation [44]. VEGF and PDGF modulate the glial response to a cortical injury [45]. Endogenous VEGF expression induces glia cell proliferation by autocrine signaling in the brain after injury [46]. VEGF also acts as a proinflammatory cytokine [47] and promotes migration and proliferation of microglia in vitro [48]. PDGF is also expressed in microglia [49] and interacts with the ionized calcium binding adaptor molecule 1 to enhance microglia motility and activation [50]. Increased calcium may also be a potential mechanism underlying the effects of angiogenesis following implantation of CG matrix after surgical brain injury. It has been demonstrated that Orai1 is upregulated in vascular smooth muscle cells (VSMC) during vascular injury and is required for nuclear factor for activated T-cell (NFAT) activity, VSMC proliferation, and neointima formation [51]. In addition, cell proliferation and cell inflammation can be regulated by calcium signaling in different types of cells [52–54]. Therefore, increased calcium might also play an important role in the enhanced angiogenesis in L + CG group.

## 5. Conclusions

Our results indicate that implantation of CG scaffolds leads to a well-developed vasculature. This study verifies the angiogenic effect of a CG used on a surgical brain trauma defect. This opens new opportunities for the clinical use of C-GAG scaffolds for tissue regeneration following brain injuries such as ischemia or trauma.
